# Development and Validation of a Prediction Model for Future Estimated Glomerular Filtration Rate in People With Type 2 Diabetes and Chronic Kidney Disease

**DOI:** 10.1001/jamanetworkopen.2023.1870

**Published:** 2023-04-05

**Authors:** Mariella Gregorich, Michael Kammer, Andreas Heinzel, Carsten Böger, Kai-Uwe Eckardt, Hiddo Lambers Heerspink, Bettina Jung, Gert Mayer, Heike Meiselbach, Matthias Schmid, Ulla T. Schultheiss, Georg Heinze, Rainer Oberbauer

**Affiliations:** 1 Center for Medical Data Science, Section for Clinical Biometrics, Medical University of Vienna, Vienna, Austria; 2 Division of Nephrology and Dialysis, Department of Internal Medicine III, Medical University of Vienna, Vienna, Austria; 3 Department of Nephrology, University of Regensburg, University Hospital Regensburg, Regensburg, Germany; 4 Department of Nephrology, Diabetology, and Rheumatology, Traunstein Hospital, Southeast Bavarian Clinics, Traunstein, Germany; 5 KfH Kidney Center Traunstein, Traunstein, Germany; 6 Department of Nephrology and Medical Intensive Care, Charité University Medicine Berlin, Berlin, Germany; 7 Department of Nephrology and Hypertension, Friedrich-Alexander University Erlangen-Nürnberg, Erlangen, Germany; 8 Department of Clinical Pharmacy and Pharmacology, University of Groningen, University Medical Centre Groningen, Groningen, the Netherlands; 9 Department of Internal Medicine IV–Nephrology and Hypertension, Medical University Innsbruck, Innsbruck, Austria; 10 Institute of Medical Biometry, Informatics, and Epidemiology, University Hospital Bonn, Bonn, Germany; 11 Institute of Genetic Epidemiology and Department of Medicine IV–Nephrology and Primary Care, Faculty of Medicine and Medical Center, University of Freiburg, Freiburg, Germany

## Abstract

**Importance:**

Type 2 diabetes increases the risk of progressive diabetic kidney disease, but reliable prediction tools that can be used in clinical practice and aid in patients’ understanding of disease progression are currently lacking.

**Objective:**

To develop and externally validate a model to predict future trajectories in estimated glomerular filtration rate (eGFR) in adults with type 2 diabetes and chronic kidney disease using data from 3 European multinational cohorts.

**Design, Setting, and Participants:**

This prognostic study used baseline and follow-up information collected between February 2010 and December 2019 from 3 prospective multinational cohort studies: PROVALID (Prospective Cohort Study in Patients with Type 2 Diabetes Mellitus for Validation of Biomarkers), GCKD (German Chronic Kidney Disease), and DIACORE (Diabetes Cohorte). A total of 4637 adult participants (aged 18-75 years) with type 2 diabetes and mildly to moderately impaired kidney function (baseline eGFR of ≥30 mL/min/1.73 m^2^) were included. Data were analyzed between June 30, 2021, and January 31, 2023.

**Main Outcomes and Measures:**

Thirteen variables readily available from routine clinical care visits (age, sex, body mass index; smoking status; hemoglobin A_1c_ [mmol/mol and percentage]; hemoglobin, and serum cholesterol levels; mean arterial pressure, urinary albumin-creatinine ratio, and intake of glucose-lowering, blood-pressure lowering, or lipid-lowering medication) were selected as predictors. Repeated eGFR measurements at baseline and follow-up visits were used as the outcome. A linear mixed-effects model for repeated eGFR measurements at study entry up to the last recorded follow-up visit (up to 5 years after baseline) was fit and externally validated.

**Results:**

Among 4637 adults with type 2 diabetes and chronic kidney disease (mean [SD] age at baseline, 63.5 [9.1] years; 2680 men [57.8%]; all of White race), 3323 participants from the PROVALID and GCKD studies (mean [SD] age at baseline, 63.2 [9.3] years; 1864 men [56.1%]) were included in the model development cohort, and 1314 participants from the DIACORE study (mean [SD] age at baseline, 64.5 [8.3] years; 816 men [62.1%]) were included in the external validation cohort, with a mean (SD) follow-up of 5.0 (0.6) years. Updating the random coefficient estimates with baseline eGFR values yielded improved predictive performance, which was particularly evident in the visual inspection of the calibration curve (calibration slope at 5 years: 1.09; 95% CI, 1.04-1.15). The prediction model had good discrimination in the validation cohort, with the lowest C statistic at 5 years after baseline (0.79; 95% CI, 0.77-0.80). The model also had predictive accuracy, with an *R*^2^ ranging from 0.70 (95% CI, 0.63-0.76) at year 1 to 0.58 (95% CI, 0.53-0.63) at year 5.

**Conclusions and Relevance:**

In this prognostic study, a reliable prediction model was developed and externally validated; the robust model was well calibrated and capable of predicting kidney function decline up to 5 years after baseline. The results and prediction model are publicly available in an accompanying web-based application, which may open the way for improved prediction of individual eGFR trajectories and disease progression.

## Key Points

**Question:**

Can routinely available data from primary care visits be used to develop and externally validate a prediction model that reliably predicts estimated glomerular filtration rate (eGFR) for upcoming follow-up visits?

**Findings:**

In this prognostic study involving 4637 adults with type 2 diabetes and chronic kidney disease, a prediction model including 13 routinely collected baseline variables based on data from 3 prospective multinational cohort studies was developed and externally validated. The model was robust, well calibrated, and capable of predicting decreases in eGFR up to 5 years after baseline.

**Meaning:**

These findings suggest that the prediction model, which is publicly available in a web-based application, may be used to improve prediction of individual eGFR trajectories and kidney function decline.

## Teaser

This prognostic study uses data from 3 European multinational cohort studies to develop and externally validate a model that can predict individual trajectories in estimated glomerular filtration rate among adults with type 2 diabetes and chronic kidney disease.

## Introduction

Chronic kidney disease (CKD) is one of the most common complications of diabetes, occurring in approximately 40% of patients with type 2 diabetes.^1,2^ Early awareness and identification of individuals at risk of rapid progression are at the forefront of CKD prevention. The disease is characterized by progressive loss of kidney function, which is assessed by sequential estimated glomerular filtration rate (eGFR) and can vary substantially between individuals. Monitoring is performed using noninvasive methods consisting of annual physical examinations, laboratory test results, and determination of eGFR. A clinically useful prediction model of future eGFR measurements based on these routinely collected clinical data may help clinicians guide and plan preventive interventions as well as improve patient understanding and patient-physician communication about the further course of kidney function decline.^3^

Previous studies^4,5^ that aimed to predict progressive loss of kidney function used an arbitrary definition to create a binary outcome based on continuously measured eGFR, limiting the accuracy of the prediction. Generally, prediction models focus on definitive kidney function end points (eg, kidney replacement therapy),^4,5,6,7,8,9,10^ even if these end points are artificially created based on repeatedly observed laboratory measurements, such as doubling of serum creatinine levels or eGFR slope-based approaches quantifying yearly loss.^11,12,13^ For example, Gurudas et al^14^ defined the split for risk classification as the latest of the first 2 eGFR measurements lower than 60 mL/min/1.73 m^2^, obtained at least 3 months apart, to predict the risk of incident stage 3 CKD. More commonly, eGFR slope-based approaches based on repeated eGFR recordings are used as proxies for annual estimated decline in kidney function.^15^ However, these end points are substantially less definitive in contrast to kidney replacement therapy and are limited by the inter- and intraparticipant variability inherent to eGFR readings as well as estimation accuracy that is dependent on the number of time points available. Despite the known ambiguity in eGFR slope estimation and the benefits of continuous monitoring of disease deterioration, prediction modeling for future eGFR values remains largely overlooked.

We sought to optimally predict eGFR values at upcoming follow-up visits conditional on baseline eGFR using linear mixed-effects modeling based on routine clinical care data from patients with type 2 diabetes and CKD. We adopted the analysis strategy outlined by Gregorich et al,^16^ in which baseline eGFR measurements are part of the outcome to be modeled and at application of the model are used to compute posterior random slopes and intercepts for the eGFR trajectory. This novel prediction model may allow patients and treating physicians to predict future kidney function by using commonly available variables, including current eGFR. We also implemented a user-friendly web-based application of the model to further facilitate the translation to clinical practice and aid in patient understanding of disease progression.

## Methods

This prognostic study used baseline and follow-up data collected between January 2010 and December 2019 from 4637 participants in 3 European multinational prospective cohort studies: PROVALID (Prospective Cohort Study in Patients with Type 2 Diabetes Mellitus for Validation of Biomarkers),^17^ GCKD (German Chronic Kidney Disease),^18^ and DIACORE (Diabetes Cohorte).^19^ Data were analyzed between June 30, 2021, and January 31, 2023. For the current study, a detailed description of the background of the study cohorts, inclusion and exclusion criteria, statistical methods describing the computation of random-effect coefficients based on baseline information for new individuals, validation procedures, and individual risk assessment of rapid kidney deterioration was previously published in the study protocol.^16^ The PROVALID, GCKD, and DIACORE studies all received approval from the responsible local institutional review boards in each participating country. Written informed consent was required for participation in all 3 studies, and the protocols and data protection strategies were also reviewed and approved by the appropriate ethics committees and data protection officers. All of these studies were conducted in accordance with the Declaration of Helsinki.^17,18,19,20^ Approval for the current study was covered by the approvals of the ethics committees of the separate study cohorts. This study followed the Transparent Reporting of a Multivariable Prediction Model for Individual Prognosis or Diagnosis (TRIPOD) reporting guideline for prognostic studies.^21^

### Study Population

#### Development Cohort

**Figure 1.**
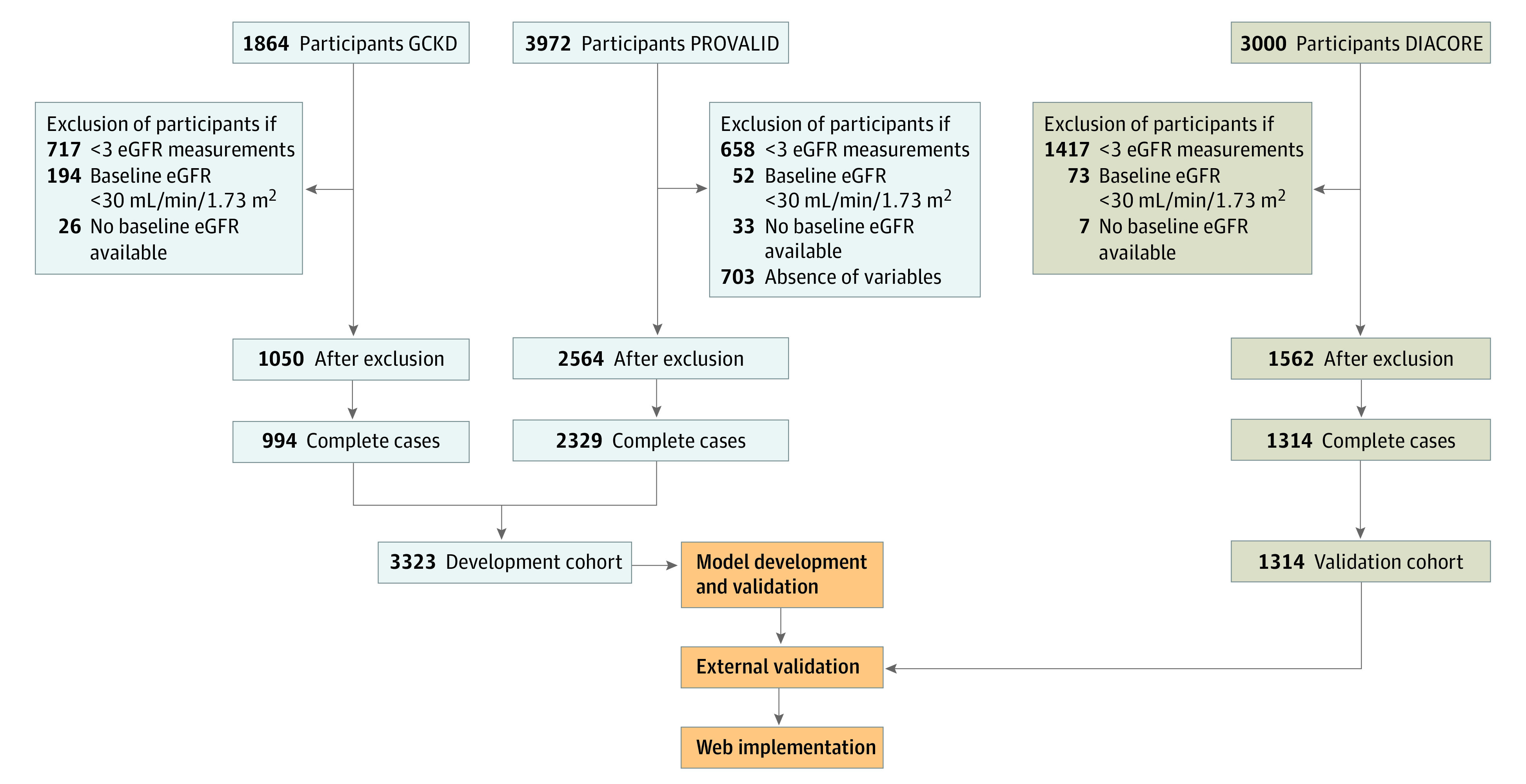
Overview of Study Design The numbers excluded sum to more than the the totals shown after exclusion because participants could be excluded based on more than 1 of the exclusion criteria listed. DIACORE indicates Diabetes Cohort; eGFR, estimated glomerular filtration rate; GCKD, German Chronic Kidney Disease; and PROVALID, Prospective Cohort Study in Patients With Type 2 Diabetes Mellitus for Validation of Biomarkers.

The development cohort comprised participants from the PROVALID^17^ and GCKD^18^ studies. All individuals underwent clinical assessment during follow-up visits (once every year in the PROVALID study and once every 2 years in the GCKD study). Eligible individuals had type 2 diabetes and White race (as stated in the database; all 3 studies had White race as an inclusion criterion because most participants in a Europe-based cohort would be White), were aged 18 to 75 years, and had 3 or more eGFR readings, at least 2 years of follow-up (to ensure stable estimation of eGFR trajectories), and mildly to moderately impaired kidney function (baseline eGFR of ≥30 mL/min/1.73 m^2^). Due to the low amount of missing data, only complete cases were considered, resulting in 3323 participants (994 people from the GCKD study and 2329 people from the PROVALID study) (Figure 1). In the initial GCKD sample, only individuals with type 2 diabetes were included. Recruitment for the PROVALID and GCKD studies started in January 2010, with a mean (SD) follow up of 4.7 (1.5) years in the PROVALID study and 5.6 (0.8) years in the GCKD study.

#### External Validation Cohort

Individuals within the DIACORE (Diabetes Cohorte) study^19,22^ comprised the external validation cohort, which constituted a population that was generally comparable with individuals from the PROVALID and GCKD studies. Criteria for patient eligibility in the validation cohort were the same as those used for the development cohort, yielding 1314 people (Figure 1). In the DIACORE study, enrollment was initiated in January 2010, with a mean (SD) follow-up of 5.1 (0.6) years.

### Variables

#### Covariates

Candidate predictor variables were selected by medical expertise and availability in routine clinical care visits and were only recorded at the individual’s first study visit (baseline). The following 13 baseline predictors were included in the model: age, sex, body mass index (BMI; calculated as weight in kilograms divided by height in meters squared), smoking status (never or ever), hemoglobin A_1c_ level (mmol/mol and percentage), hemoglobin level (g/dL), serum cholesterol level (mg/dL), mean arterial pressure, urinary albumin-creatinine ratio (mg/g), and intake of glucose-lowering, blood pressure–lowering, or lipid-lowering medication (yes or no). Information about the composition of medication classes is provided in eAppendix 9 in Supplement 1. Time of eGFR assessment since the baseline visit was also included as a predictor in the model. The urinary albumin-creatinine ratio was log_2_ transformed to reduce the impact of highly influential points.

#### Outcome

The primary outcome was repeated eGFR measurements recorded at baseline and follow-up visits. The eGFR values were calculated using the Chronic Kidney Disease Epidemiology Collaboration equation from 2021, which includes the person’s sex, age, and serum creatinine level.^23,24^

### Statistical Analysis

#### Model Specification

To account for within-participant and between-participant variability, we modeled repeated eGFR measurements over time using a multivariable linear mixed-effects model. We used fixed effects for each candidate predictor and also included an interaction (product) term of each predictor with time.^25^ Furthermore, we included random intercepts and a random coefficient for time (random slopes) for individuals and used an unstructured covariance-variance matrix. In addition, we accounted for country-specific differences in eGFR levels by nesting the individual-specific random intercepts within countries. Conditional and marginal *R*^2^ values were computed for the mixed-effects model, corresponding to explained variation including the random effects (conditional *R*^2^ values) or excluding the random effects (marginal *R*^2^ values).

#### Computation of Individual-Specific Random Effects by Baseline eGFR

In our modeling approach, baseline eGFR values were included in the response vector, and response profiles were modeled by the predictor variables.^26^ In this way, baseline eGFR values contributed to estimates of random variability (magnitude of measurement error) and changes in eGFR over time. The model can be used to predict individual-specific response profiles, including baseline and follow-up values; however, if baseline values are already known, they can be incorporated into the prediction to increase the predictive accuracy for follow-up values (model equations are available in eAppendix 4 in Supplement 1). For these reasons, we did not model the repeated measurements of eGFR dynamically but only used the first available eGFR measurement (at baseline) of a new individual to obtain updated individual-specific random effects of the model that would allow more precise predictions of eGFR.

#### Internal-External and External Validation

We used internal-external validation to evaluate the predictive ability of the model in the development cohort using country as a nonrandom splitting unit, and we computed 95% CIs by the bootstrap method.^27^ Calibration slopes, predicted *R*^2^ values, and C statistics (eAppendix 5 in Supplement 1) were determined based on predictions obtained after updating the random coefficient estimates with baseline eGFR measurements. Using the same performance measures, we evaluated the predictive capability of the final model in the external validation cohort. Moreover, calibration of the final model in the external cohort was graphically assessed by plotting the observed eGFR values against their predictions for each follow-up year. Details of the validation procedure and the performance measures can be found in eAppendix 5 and eAppendix 8 in Supplement 1.

#### Estimation of eGFR Slopes and Predicted Probabilities of Rapid Decline

From the fitted and validated model, expected values and variances of individual-specific eGFR slopes of kidney function were obtained by computing the derivative of the model equation with respect to time and inserting the baseline eGFR value and predictor values into the derivative. The probability of rapid progression was then computed by normal approximation using an eGFR threshold of −3 mL/min/1.73 m^2^ per year to differentiate between stable and rapid progression. Specifically, the probability of rapid progression was reported as the value of the predictive distribution function of the estimated slope at the chosen threshold.

All analyses were performed using R software, version 4.2.1 (R Foundation for Statistical Computing). The shiny package for R software was used for implementation of the web application.

## Results

### Patient Characteristics and Measurements

Among 4637 adults with type 2 diabetes and CKD (mean [SD] age at baseline, 63.5 [9.1] years; 2680 men [57.8%] and 1957 women [42.2%]; all of White race), 3323 individuals were included in the development cohort (mean [SD] age at baseline, 63.2 [9.3] years; 1864 men [56.1%] and 1459 women [43.9%]), and 1314 individuals were included in the external validation cohort (mean [SD] age at baseline, 64.5 [8.3] years; 816 men [62.1%] and 498 women [37.9%]). Additional baseline characteristics stratified by study of origin (GCKD, PROVALID, or DIACORE) are shown in Table 1. The mean (SD) baseline eGFR was 52.4 (15.6) mL/min/1.73 m^2^ in the GCKD cohort, 89.6 (19.8) mL/min/1.73 m^2^ in the PROVALID cohort, and 81.2 (16.7) mL/min/1.73 m^2^ in the DIACORE cohort. Pearson correlation analysis did not reveal an association between any pair of independent variables (eAppendix 3 in Supplement 1). The median rate of eGFR decline, estimated using individual-specific linear regression analysis, was similar across cohorts (PROVALID: median [IQR], −1.45 [−3.51 to −0.23] mL/min/1.73 m^2^ per year; GCKD: median [IQR], −1.43 [−3.10 to 0.01] mL/min/1.73 m^2^ per year; DIACORE: median [IQR], −1.28 [−2.87 to −0.12] mL/min/1.73 m^2^ per year). Availability of data points per year of follow-up and eGFR distributions across years are shown in eAppendix 1 and eAppendix 2 in Supplement 1.

**Table 1.** Baseline Characteristics of Development and External Validation Cohorts

Characteristic	Participants, No. (%)		
	Development cohort		External validation cohort
	GCKD study (n = 994)	PROVALID study (n = 2329)	DIACORE study (n = 1314)
**Demographic and clinical characteristics**			
Age, mean (SD), y	64.0 (8.3)	62.8 (9.7)	64.5 (8.3)
Sex			
Female	335 (33.7)	1124 (48.3)	498 (37.9)
Male	659 (66.3)	1205 (51.7)	816 (62.1)
Ever smoked	618 (62.2)	1176 (50.5)	584 (44.4)
BMI, mean (SD)	32.3 (5.8)	31.0 (5.3)	31.0 (5.3)
Medication			
Glucose-lowering	785 (79.0)	2136 (91.7)	1141 (86.8)
Blood pressure–lowering	975 (98.1)	1849 (79.4)	1052 (80.1)
Lipid-lowering	639 (64.3)	1404 (60.3)	640 (48.7)
**Laboratory measurements**			
Mean arterial pressure, mean (SD)	98.5 (12.4)	99.1 (10.7)	97.3 (10.8)
Blood pressure, mean (SD), mm Hg			
Systolic	141.3 (19.8)	136.9 (16.8)	139.1 (17.5)
Diastolic	77.1 (11.2)	80.1 (9.9)	76.3 (9.8)
HbA_1c_, mean (SD), mmol/mol	55.6 (11.2)	52.8 (12.6)	51.0 (10.8)
HbA_1c_, mean (SD), %	7.3 (1.0)	7.0 (1.2)	6.8 (1.0)
Serum cholesterol, mean (SD), mg/dL	200.1 (45.9)	186.2 (46.8)	203.5 (42.4)
Hemoglobin, mean (SD), g/dL	13.7 (1.6)	13.9 (1.5)	14.4 (1.2)
UACR, median (IQR), mg/g	37.1 (8.0-280.1)	9.6 (4.3-26.5)	9.0 (4.5-24.4)
Log_2_ UACR, mean (SD), mg/g	5.6 (3.0)	3.4 (2.5)	3.6 (1.9)
eGFR, mean (SD), mL/min/1.73 m^2^	52.4 (15.6)	89.6 (19.8)	81.2 (16.7)

Abbreviations: BMI, body mass index (calculated as weight in kilograms divided by height in meters squared); DIACORE, Diabetes Cohort; eGFR, estimated glomerular filtration rate; GCKD, German Chronic Kidney Disease; HbA_1c_, hemoglobin A_1c_; PROVALID, Prospective Cohort Study in Patients With Type 2 Diabetes Mellitus for Validation of Biomarkers; UACR, urine albumin-creatinine ratio.

### Model Development and Internal-External Validation

Before updating the random effects coefficients using baseline eGFR values, the overall conditional *R*^2^ was 0.90, and the marginal *R*^2^ was 0.20. The most important predictor was age, with a decrease in marginal *R*^2^ of 0.10 (95% CI, 0.08-0.10) (eAppendix 7 in Supplement 1). The internally-externally cross-validated performance measures of discrimination, calibration, and model fit, stratified by follow-up year, are provided in Table 2. The time-specific predicted *R*^2^ values ranged from 0.74 (95% CI, 0.59-0.84) at year 1 to 0.47 (95% CI, 0.25-0.68) at year 5, and the C statistic ranged from 0.84 (95% CI, 0.78-0.88) at year 1 to 0.75 (95% CI, 0.67-0.82) at year 5, with lower values observed after the first follow-up year.

**Table 2.** Measures of Prediction Model Performance and Validity

Follow-up year	Performance measure (95% CI)		
	*R*^2^ value	C statistic	Calibration slope
**Cross-validated**			
1	0.74 (0.59-0.84)	0.84 (0.78-0.88)	1.05 (0.88-1.16)
2	0.63 (0.50-0.77)	0.80 (0.76-0.85)	1.01 (0.85-1.18)
3	0.59 (0.42-0.76)	0.80 (0.74-0.85)	0.97 (0.74-1.20)
4	0.55 (0.39-0.73)	0.77 (0.71-0.83)	0.98 (0.72-1.22)
5	0.47 (0.25-0.68)	0.75 (0.67-0.82)	0.88 (0.60-1.12)
**Externally validated**			
1^a^	NA	NA	NA
2	0.70 (0.63-0.76)	0.83 (0.81-0.85)	1.10 (1.02-1.17)
3	0.64 (0.58-0.70)	0.82 (0.80-0.83)	1.11 (1.06-1.16)
4	0.65 (0.58-0.72)	0.81 (0.79-0.83)	1.13 (1.06-1.21)
5	0.58 (0.53-0.63)	0.79 (0.77-0.80)	1.09 (1.04-1.15)

Abbreviation: NA, not available.

^a^ Estimated glomerular filtration rate measurements were not obtained in the first follow-up year of the DIACORE (Diabetes Cohort) study.

**Figure 2.**
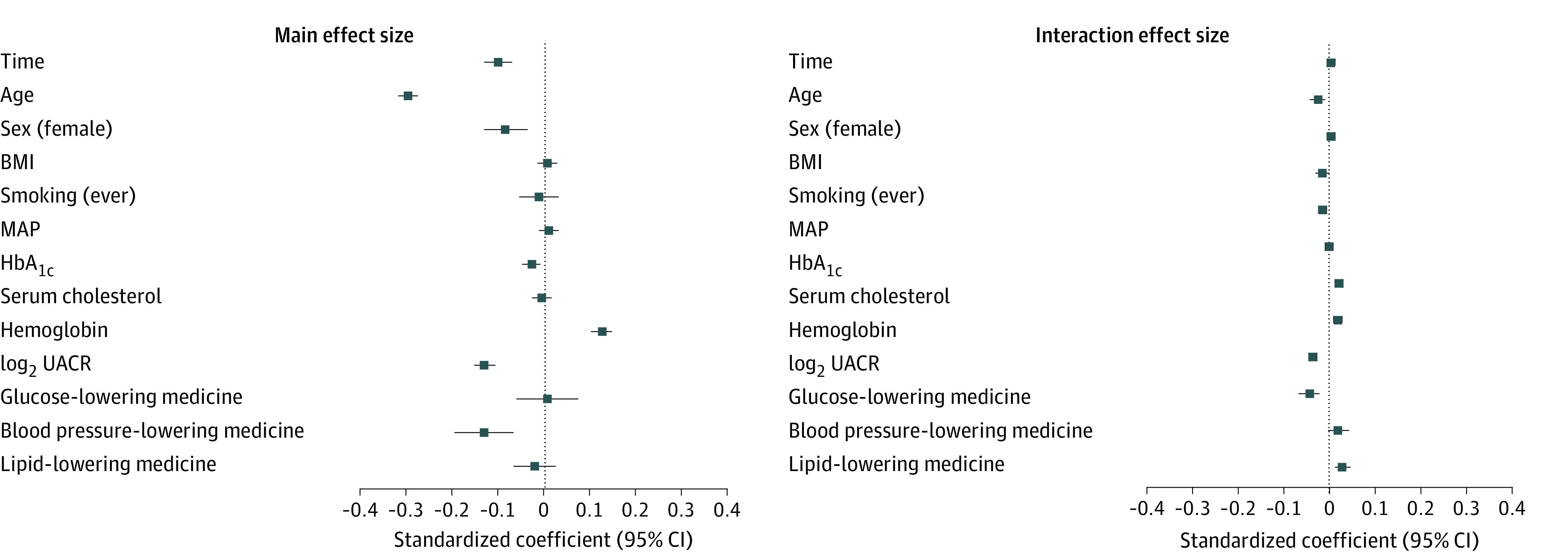
Standardized Regression Coefficients of the Model Standardized model coefficients were used for continuous variables, and unstandardized model coefficients were used for binary variables. Each coefficient represents the difference in predicted outcome per difference of 1 SD for a continuous predictor or when comparing the 2 levels of a binary predictor. Whiskers represent 95% CIs. BMI indicates body mass index (calculated as weight in kilograms divided by height in meters squared); MAP, mean arterial pressure; HbA_1c_, hemoglobin A_1c_; and UACR, urine albumin-creatinine ratio.

The standardized coefficients of the predictors’ main effect size estimates and their interactions with time are shown in Figure 2. All variables except BMI, smoking status, mean arterial pressure, serum cholesterol, glucose-lowering medication, and lipid-lowering medication were associated with significant decreases in eGFR, with age having the greatest reduction (estimate, −0.30; 95% CI, −0.32 to −0.28). However, the interaction effect of these 6 variables was associated with significant decreases in eGFR (Figure 2). The magnitude of the standardized interaction effects was low compared with the main effect size estimates. Unstandardized coefficients and estimated variance parameters are reported in eAppendix 6 in Supplement 1.

### External Validation

**Figure 3.**
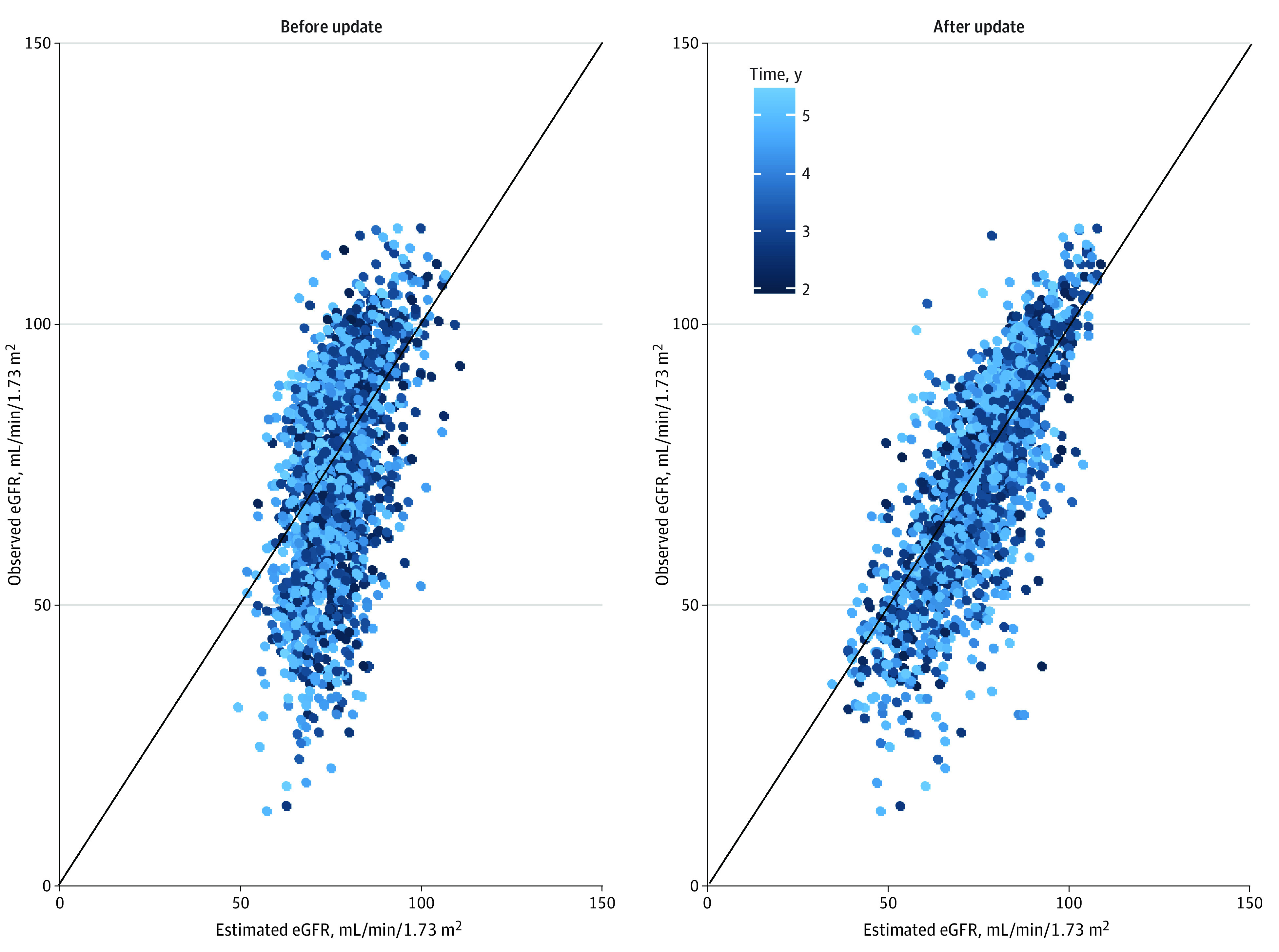
Change in Calibration When Updating Random Coefficients by Baseline Estimated Glomerular Filtration Rate (eGFR) Comparison between predictions before and after updating the random coefficient estimates for baseline eGFR measurements from the validation cohort. Later time points are indicated by lighter coloring. The 45° solid line starting at 0 indicates perfect agreement between the estimated and observed eGFR values.

In the external validation cohort, eGFR measurements were available from year 2 to year 5 after baseline. The associated performance measures are reported in Table 2. To illustrate the dominant role of baseline eGFR, we contrasted the calibration of our model before (left panel in Figure 3) and after (right panel in Figure 3) the predictions. Overall, updating the random effects with baseline eGFR yielded excellent agreement between the predicted and observed eGFR values in the validation cohort, especially for early follow-up years. Furthermore, the externally validated *R*^2^ ranged from 0.70 (95% CI, 0.63-0.76) at year 1 to 0.58 (95% CI, 0.53-0.63) at year 5. The C statistic ranged from 0.83 (95% CI, 0.81-0.85) at year 1 to 0.79 (95% CI, 0.77-0.80) at year 5. The calibration slope was highest at follow-up year 4 (1.13; 95% CI, 1.06-1.21) and lowest at follow-up year 5 (1.09; 95% CI, 1.04-1.15), suggesting stable predictive capabilities of the model in unseen individuals (ie, individuals the model has not been trained on). The assessment of time-specific calibration slopes revealed an almost perfect calibration at up to 4 years after baseline and only minimal shrinkage at 5 years after baseline (Figure 3).

### Web-Based Application

The prediction model for an individual’s eGFR at future follow-up time points, visualization of model results, and risk assessment for rapid progression was implemented as an online risk calculator.^28^ The code for model development and validation without the data, and the code for the shiny app (for R software) are openly available in the GitHub repository at BEAt-DKD_PredeGFR.^29^

## Discussion

In this prognostic study, we developed a robust and well-calibrated prediction model for kidney function decline that was able to predict future eGFR values after baseline assessment in adults with type 2 diabetes and early to moderately progressed CKD using 2 large European study cohorts. External validation of the model based on the availability of routinely collected demographic and clinical data revealed good predictive performance of kidney function decline up to 5 years after baseline assessment. We addressed several common methodological limitations of prediction modeling studies, such as the lack of external validation, to ensure generalizability of application to unseen cohorts^7,10,30^ and inclusion of predictors that are not routinely available in primary clinical care visits (eg, genetic information and serum biomarkers).^15,31,32^ For instance, Jiang et al^31^ considered genetic covariates in addition to traditional covariates when investigating the association between known genetic variants and eGFR trajectories. However, genome-wide genotyping is not available in daily clinical practice and hence is not suitable for general use. Furthermore, the incremental usefulness of molecular biomarkers in addition to traditional clinical predictors for improved prediction is still under investigation.^33,34^ Therefore, we restricted the model to data obtained in primary clinical care visits among individuals with CKD and type 2 diabetes.

Only 3 studies^33,35,36^ were found that focused on the development of a rigorous prediction model using a sequence of repeated eGFR values as the outcome vector of interest. Khazaee et al^35^ developed and validated an artificial neural network–based prediction model in a single-center study cohort to predict future eGFR measurements for follow-up visits after kidney transplant. The prediction model was further used to derive the risk of kidney function decline among transplant recipients. However, in contrast to our study, kidney function was assessed in transplanted kidneys. Similarly, the sequence of repeated eGFR values was also used as the outcome of interest in studies by Mayer et al^36^ and Heinzel et al.^33^ However, the main objective of these 2 studies was to assess the incremental predictive utility of a panel of protein measurements for kidney failure; therefore, their models would not be applicable in the primary care setting, nor have they been validated externally.

### Strengths and Limitations

This study has several strengths. First, the study uses baseline eGFR values as part of the outcome vector and not as a covariate. Baseline eGFR measurements are subject to the same measurement errors as follow-up eGFR measurements; by including baseline measurements in the outcome vector, we can account for and explicitly estimate these variabilities in the model. The mixed-effects modeling method provides a straightforward approach to consider baseline values for predictions by way of updating random effects, as observed in this study. Second, rather than selecting variables based on observed associations with the outcome, we included a prespecified set of predictors in our model based on their availability during routine primary care visits, which has repeatedly been proposed as the preferred approach to model building.^37,38^ Third, we included these predictors, as well as their interactions with follow-up time, as main outcomes to predict changes in kidney function, even if this inclusion only marginally increased predictive accuracy. Fourth, the model was internally-externally validated with 1000 bootstrap samples to obtain 95% CIs for the performance measures in the development cohort, yielding good overall predictive performance and model fit. Rigorous external validation in a large cohort further confirmed the generalizability of the model and its stable predictive capabilities in terms of calibration and discrimination, suggesting an ability to accurately predict future kidney function solely based on data from routine primary care visits.

Fifth, the web-based application provides a user-friendly prediction tool that can be used to identify patients with high risk and rapid decline in kidney function for recruitment in prospective clinical studies. Such assistance with patient selection can improve the quality of clinical trials by ensuring that more patients with relevant conditions and fewer patients with nonrelevant conditions are selected, thereby reducing the cost and duration of clinical trials. However, before the model can be used as a basis for treatment decisions, it should undergo further prospective evaluation of the benefits and harms of its application in an impact study.^39^ In addition, the web-based application may help to combat the current lack of awareness regarding the process and speed of kidney function decline in CKD by displaying the potential course of disease.

This study also has limitations. First, all 3 of the prospectively planned large-scale cohort studies were conducted in European countries, which is the reason we used the Chronic Kidney Disease Epidemiology Collaboration equation to estimate GFR.^24^ Future research could strengthen the validity of this prediction model by including cohorts from additional countries. Second, creatinine assays were not standardized across cohorts, which is a known issue that can introduce inherent variability in clinical laboratory serum creatinine measurements; this issue was taken into account via the estimation of country-specific random intercept terms in the model. Third, the availability of data points grew sparser at later time points (eAppendix 2 in Supplement 1); hence, the accuracy of the predictions decreased at later time points. Because of this issue, we limited the applicability of the web-based implementation of the prediction model to a maximal time span of 5 years after the baseline examination to ensure reliable predictions of eGFR. Fourth, the 3 prospective studies included in our analyses started recruitment in 2010 and had a mean follow-up of approximately 5 years. Thus, medications that were approved for the treatment of CKD by regulatory authorities shortly before or after 2010 and 2011 (such as sodium-glucose cotransporter 2 inhibitors, nonsteroidal mineralocorticoid receptor antagonists, and glucagon-like peptide 1 receptor agonists) were either not available or not routinely used throughout the study periods. Renin angiotensin aldosterone system blockade was quantitatively available only during the PROVALID study and was therefore not included in the model.

## Conclusions

This prognostic study used a linear mixed-effects model to predict eGFR trajectories among adults with type 2 diabetes and CKD; this model naturally circumvented the inherent issues related to eGFR slope estimation and fully incorporated the observed data into model estimation. Despite its complexity, the prediction model was robust, well calibrated, and suitable for implementation in a web-based application, revealing the potential of a publicly available online tool that can be used by patients, caregivers, and primary health care professionals to predict individual eGFR trajectories and disease progression up to 5 years after baseline.

## Notes

Supplement 1. **eAppendix 1.** Data Availability **eAppendix 2.** Distribution of Data Points Across Follow-up **eAppendix 3.** Correlation Between Predictors **eAppendix 4.** Prediction Model Equation **eAppendix 5.** Extended Statistical Methods **eAppendix 6.** Fixed-Effect Coefficients and Their Time Interactions in the Prediction Model **eAppendix 7.** Partial *R*^2^ Coefficients **eAppendix 8.** Model Comparison **eAppendix 9.** Composition of Medication Classes
eReferences

Supplement 2.
Data Sharing Statement
