# Supplementary material for: Development and Validation of a Prediction Model for Future Estimated Glomerular Filtration Rate in People With Type 2 Diabetes and Chronic Kidney Disease
Source: JAMA Netw Open. 2023 Apr 5;6(4):e231870. doi: 10.1001/jamanetworkopen.2023.1870 (PMC10077108; doi:10.1001/jamanetworkopen.2023.1870)
Supplement: Supplement 1. — eAppendix 1. Data Availability eAppendix 2. Distribution of Data Points Across Follow-up eAppendix 3. Correlation Between Predictors eAppendix 4. Prediction Model Equation eAppendix 5. Extended Statistical Methods eAppendix 6. Fixed-Effect Coefficients and Their Time Interactions in the Prediction Model eAppendix 7. Partial R2 Coefficients eAppendix 8. Model Comparison eAppendix 9. Composition of Medication Classes eReferences [file jamanetwopen-e231870-s001.pdf]

Supplementary Online Content

Gregorich M, Kammer M, Heinzel A, et al; BEAt-DKD Consortium. Development and validation of a prediction model for future estimated glomerular filtration rate in people with type 2 diabetes and chronic kidney disease. *JAMA Netw Open*. 2023;6(4):e231870. doi:10.1001/jamanetworkopen.2023.1870

eAppendix 1. Data Availability

eAppendix 2. Distribution of Data Points Across Follow-up

eAppendix 3. Correlation Between Predictors

eAppendix 4. Prediction Model Equation

eAppendix 5. Extended Statistical Methods

eAppendix 6. Fixed-Effect Coefficients and Their Time Interactions in the Prediction Model

eAppendix 7. Partial R^2 Coefficients

eAppendix 8. Model Comparison

eAppendix 9. Composition of Medication Classes

eReferences

This supplemental material has been provided by the authors to give readers additional information about their work.

© 2023 Gregorich M et al. *JAMA Network Open*.

eAppendix 1. Data Availability

Due to the small proportion of missing values across the three study cohorts, a complete cases analysis was carried out. Below is a supplementary table 1 with the baseline patient characteristics along with an indicator of proportional missingness from the full development and validation cohort before the mission of individuals with missing values.

Table S1. Distribution of baseline characteristics in the development and validation cohorts before complete-case analysis

	**Development cohort**				**Validation cohort**	
Variable	GCKD	NA (%)	PROVALID	NA (%)	DIACORE	NA (%)
N	1050		2564		1562	
**Demographic variables**						
Age, years	64.1 ± 8.2	0.0	63.0 ± 9.7	0.0	64.5 ± 8.4	0.0
Sex, female	354 (33.7)	0.0	1224 (47.7)	0.0	592 (37.9)	0.0
Sex, male	696 (66.3)	0.0	1340 (47.8)	0.0	970 (62.1)	0.0
Smoking, ever	651 (62.0)	0.0	1245 (50.6)	4.1	680 (43.5)	0.0
BMI, kg/m^2	32.3 ± 5.7	1.2	31.0 ± 5.3	0.4	31.1 ± 5.3	0.3
**Laboratory measurements**						
Mean arterial pressure	98.5 ± 12.4	0.5	99.2 ± 10.7	0.2	97.2 ± 10.7	0.1
Systolic blood pressure, mmHg	141.3 ± 19.7	0.5	137.3 ± 17.0	0.2	138.8 ± 17.5	0.1
Diastolic blood pressure, mmHg	77.1 ± 11.2	0.5	80.2 ± 9.9	0.2	76.3 ± 9.8	0.1
HbA_{1c}, %	7.2 ± 1.0	1.3	7.0 ± 1.2	0.4	6.8 ± 1.0	0.1
HbA_{1c}, mmol/mol	55.6 ± 11.2	1.3	52.7 ± 12.5	0.4	51.1 ± 10.8	0.1
Serum Cholesterol, mg/dl	199.9 ± 46.1	0.1	185.3 ± 46.8	0.3	203.1 ± 41.8	0.0
Hemoglobin, g/dl	13.7 ± 1.6	1.5	13.9 ± 1.5	3.5	14.4 ± 1.2	13.1
UACR, mg/g	37.1 [8.0, 283.0]	2.4	9.3 [4.3, 26.3]	1.9	9.0 [4.5, 24.4]	2.6
Log2 UACR, mg/g	5.6 ± 3.0	2.4	3.4 ± 2.5	1.9	3.6 ± 2.0	2.6
eGFR, ml/min/1.73m^2	52.5 ± 15.6	0.0	89.6 ± 19.5	0.0	81.4 ± 16.9	0.0
**Medication intake**						
Glucose-lowering	833 (79.3)	0.0	2348 (91.6)	0.0	1349 (86.4)	0.0
Blood pressure-lowering	1030 (98.1)	0.0	2035 (79.4)	0.0	1238 (79.3)	0.0
Lipid-lowering	677 (64.5)	0.0	1550 (60.6)	0.0	764 (48.9)	0.0

N, sample size; BMI, body-mass index; HbA1c, hemoglobin A1c; UACR, urine albumin-creatinine ratio; eGFR, estimated glomerular filtration rate; NA, not available.
*Normal distributed continuous variables are expressed as mean ± standard deviation, otherwise as median [interquartile range]. Binary variables are stated as number of occurrence (percentage).

© 2023 Gregorich M et al. *JAMA Network Open*.

eAppendix 2. Distribution of Data Points Across Follow-up

Figure S1 and Table S1 show the distribution of the data points over the substantial follow-up period of 8 years per study cohort (GCKD, PROVALID and DIACORE). However, there were hardly any data points left in the 8th year of follow-up. In the DIACORE study there were only two subjects for whom the 8th year follow-up was available, which is why only a small black line in the 8th year is shown in Figure S1. GCKD was collected every two years, hence most observations can be found in even follow- up years. Only individuals who could not be examined in the planned follow-up year for various reasons were surveyed in the following year. Although two year follow-up examinations were also scheduled for DIACORE, the actual examination does not seem to be as regular as in GCKD.

At baseline, 42.7% of the development cohort were in CKD stage 1, 27.1% in CKD stage 2 and 30.2% in CKD stage 3. In the validation cohort, 36.3% were in CKD stage 1, 51.4% in stage 2 and 12.3% in stage 3.

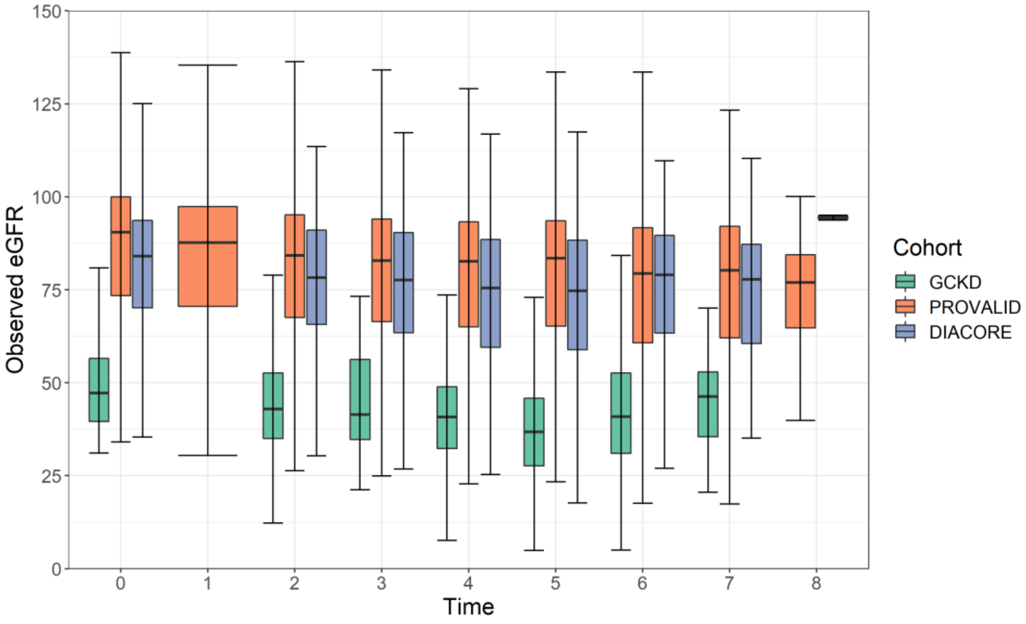

Figure S1. Distribution of eGFR values across time points stratified by the three study cohorts

A more detailed list of the numbers per year and cohort is given in Table S1. PROVALID is divided into the included countries namely Austria (AT), Hungary (HU), Poland (PL) and the United Kingdom (UK). The Netherlands is also a participating country in PROVALID, but had to be excluded due to high rates of incomplete predictors. Hemoglobin had a missingness percentage of 90.5%, smoking status of 99.6% and BMI of 41.9%. GCKD is a German study cohort. Table S1 shows that PROVALID represents a much larger part of the development cohort than GCKD.

Table S2. Number of individuals across time points stratified by the three study cohorts

	Time of follow-up eGFR assessment								
**Cohort**	0	1	2	3	4	5	6	7	8
PROVALID	2323	2148	2218	1784	1749	1097	761	258	20
AT	523	501	512	446	401	307	231	47	20
HU	988	873	970	874	784	542	451	206	0
PL	493	477	428	286	269	116	5	1	0
UK	325	301	313	181	300	136	77	5	0
GCKD (GE)	994	0	918	48	823	38	745	21	0
DIACORE	1314	0	346	644	328	671	141	47	2

As an overview of the observed eGFR curves in the study cohorts, 50 randomly selected individuals per study cohort were selected and their eGFR over time visualized in Figure S2.

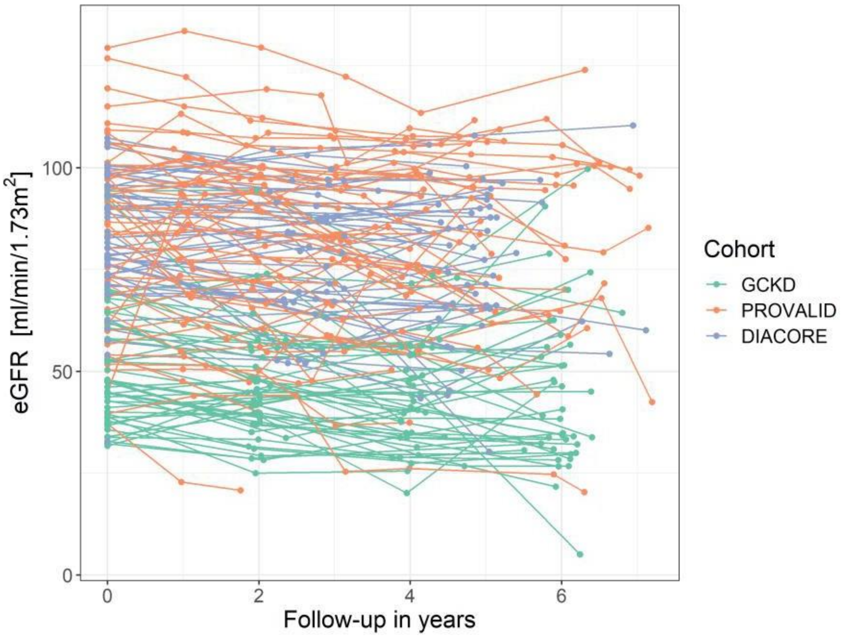

Figure S2. The observed eGFR trajectory of a random subset of individuals of each study cohort

© 2023 Gregorich M et al. *JAMA Network Open*.

eAppendix 3. Correlation Between Predictors

Spearman correlation analysis revealed no strong associations between the final predictors included in the model. The correlation heatmap is illustrated in Figure S3, with correlation ranging between -0.34 and 0.21.

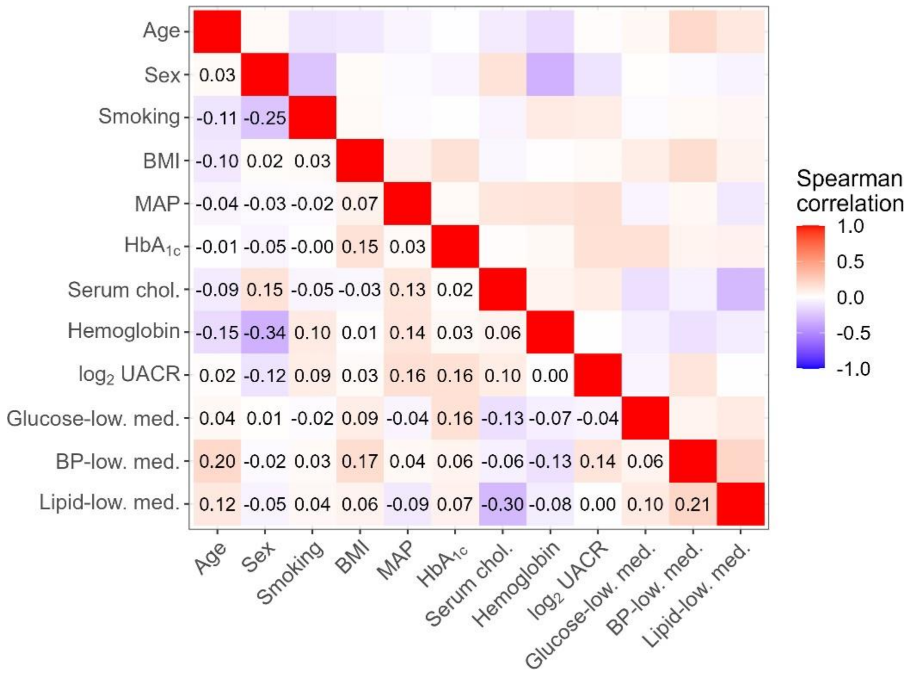

Figure S3. Spearman correlation matrix of the demographic, laboratory and medication information in the development cohort

© 2023 Gregorich M et al. *JAMA Network Open*.

eAppendix 4. Prediction Model Equation

The following predictors were included into the prediction model: time of follow-up, age, sex (female/male), smoking status (never/ever), BMI (kg/m), haemoglobin (g/dl), serum cholesterol (mg/dl), UACR (mg/g), mean arterial pressure, blood pressure-lowering medication, glucose-lowering medication and lipid-lowering medication.

The prior prediction $\hat{Y}_{it} := \widehat{\mathrm{eGFR}}_{\mathrm{it}}$ for the new subject i at time t can be obtained by

$$\begin{aligned}\hat{Y}_{\mathrm{it}} = \ & 112.610 - 1.95 * \mathrm{X_{time}} - 0.816 * X_{age} - 1.250 * [X_{sex=female}] + 0.020 * X_{BMI} + 0.233 * [X_{smoking=ever}] + 0.072 \\ & * X_{MAP} - 0.050 * X_{HbA_{1C}} - 0.017 * X_{SerumCholesterol} + 1.777 * X_{hemoglobin} - 0.735 * X_{log2UACR} + 1.611 \\ & * X_{MedDM} - 3.837 * X_{MedBP} - 1.400 * X_{MedLipid} + X_{time} \\ & * (0.004 * X_{age} - 0.331 * [X_{sex=female}] + 0.005 * X_{BMI} - 0.206 * [X_{smoking=ever}] - 0.019 * X_{MAP} \\ & - 0.003 * X_{HbA_{1C}} + 0.005 * X_{SerumCholesterol} + 0.141 * X_{hemoglobin} - 0.169 * X_{log2UACR} - 0.550 * X_{MedDM} \\ & + 0.207 * X_{MedBP} + 0.349 * X_{MedLipid})\end{aligned}$$

The updated prediction $\tilde{\mathrm{Y}}_{\mathrm{it}}$ for subject i at time t can then be computed after model fitting by estimating best linear unbiased predictors (BLUPs) of the random coefficients using the prior predictions $\hat{Y}_{\mathrm{it}}$ and the actual observed eGFR measurements at baseline $\mathrm{Y_{i0}}$

$$\hat{\mathrm{b}}_{0\mathrm{i}} = \frac{\sigma_0^2(\mathrm{Y_{i0}} - \hat{Y}_{i0})}{\sigma_0^2 + \sigma_\varepsilon^2}, \quad \text{and} \quad \hat{\mathrm{b}}_{1\mathrm{i}} = \frac{\sigma_{01}\hat{\mathrm{b}}_{0\mathrm{i}}}{\sigma_0^2}$$

with σ_0^2, σ_1^2 *and* σ_{01} denoting the diagonal and off-diagonal elements of the covariance matrix G of the random coefficients, respectively [1,2].

$$G = \begin{bmatrix} \sigma_0^2 & \sigma_{01}^2 \\ \sigma_{01}^2 & \sigma_1^2 \end{bmatrix} = \begin{bmatrix} 203.87 & -2.20 \\ -2.20 & 3.19 \end{bmatrix}$$

Plugging the updated random coefficients into

$$\tilde{\mathrm{Y}}_{\mathrm{it}} = \hat{\mathrm{Y}}_{\mathrm{it}} + \hat{\mathrm{b}}_{0\mathrm{i}} + \hat{\mathrm{b}}_{1\mathrm{i}} * X_{time}$$

then yields the updated predictions of eGFR for individual i at follow-up t.
The variance of $\tilde{Y}_{i\,t}$ is given by

$$V_{ti} = V_{oi} + (1\ t)G_{oi}(1\ t)' + \sigma_\epsilon^2$$

where V_{oi} denotes the variance of the fixed effects. G_{oi} is the conditional covariance matrix of the random effects given the individual baseline eGFR Y_{i0} with the diagonal entries

$$\sigma_{0|oi}^2 = \left(\frac{\sigma_0^2}{\sigma_0^2 + \sigma_\epsilon^2}\right)^2 V_{oi}$$

© 2023 Gregorich M et al. *JAMA Network Open*.

$$\sigma_{1|oi}^2 = \left[1 - \frac{\sigma_{01}^2}{\sigma_0^2 + \sigma_1^2}\right] \sigma_1^2$$

and the off-diagonal entries

$$\sigma_{01|oi}^2 = \frac{\sigma_{01}^2}{\sigma_1^2}$$

© 2023 Gregorich M et al. *JAMA Network Open*.

eAppendix 5. Extended Statistical Methods

This section provides further details on the statistical methods.

Internal-external validation was performed to determine the predictive performance of the model in the development cohort [3]. Specifically, at each of 1000 total iterations, we drew a bootstrap sample from each country with replacement and pooled all those country-specific samples. We then retrained the model with the bootstrapped data from all countries but one. Subsequently, we tested the predictive capabilities of the prediction model equation with the random effect coefficients updated by the baseline eGFR on the left-out country sample computing C statistics, the calibration slopes and R^2 values for each year of follow-up. During this procedure, only country-specific test samples with at least 100 individuals at a certain follow up time were used to assess performance during cross- validation due to the heterogeneity of sample size across follow-up and countries (see Supplementary Table S1) [4-6]. For example, participants in the GCKD study were examined every two years and only a few participants were surveyed in odd follow-up years who could not participate in the previous year due to time or organizational reasons, resulting in a test cohort for such years of fewer than 20 individuals.

Calibration slopes (CS) were computed using linear regression with the observed and predicted eGFR values as dependent and independent variables. The C statistic for a continuous outcome is derived from the Kendall tau-b coefficient τ_b and can be computed by

$$C = \frac{\tau_b}{2} + 0.5$$

to assess the discriminative capability of the model. Equivalent to the C statistic for binary outcomes, the C statistic for continuous outcomes can be interpreted as the probability that a randomly selected patient with a higher observed eGFR will also receive a higher predicted eGFR than a patient with a lower observed eGFR. The C statistic ranges from 0 to 1, where 1 means perfect discrimination, a value of 0.5 means a model no better than chance, and under 0.5 means that predictions are discordant with observed outcome values. R^2 is the square of the correlation coefficient of predicted and observed eGFR values.

In addition to the model development described in the main manuscript, we also used restricted cubic splines with 3-5 degrees of freedom to assess non-linearity for continuous predictors but noticed no substantial improvement over linearity in terms of R^2 that would warrant the higher model complexity. We did not conduct variable selection or regularization as the set of predictors and the modelling strategy were prespecified and the dataset deemed large enough to make additional shrinkage unnecessary.

© 2023 Gregorich M et al. *JAMA Network Open*.

eAppendix 6. Fixed-Effect Coefficients and Their Time Interactions in the Prediction Model

Table S3. Fixed effect coefficients of the multivariable linear mixed model for kidney function decline

Variable	Baseline		Slope	
	Effect	95% CI	Effect	95% CI
Constant	112.610	(97.509, 127.711)	-1.950	(-3.547, -0.352)
Age, years	-0.816	(-0.877, -0.756)	0.004	(-0.006, 0.015)
Sex, female	-1.250	(-2.447, -0.053)	-0.331	(-0.539, -0.122)
BMI	0.020	(-0.082, 0.121)	0.005	(-0.012, 0.023)
Smoking, never/ever	0.233	(-0.870, 1.337)	-0.206	(-0.397, -0.014)
MAP	0.072	(0.022, 0.121)	-0.019	(-0.028, -0.011)
HbA_{1c}	-0.050	(-0.095, -0.005)	-0.003	(-0.011, 0.005)
Serum cholesterol	-0.017	(-0.029, -0.005)	0.005	(0.003, 0.007)
Hemoglobin	1.777	(1.387, 2.168)	0.141	(0.073, 0.210)
$\log_2$ UACR	-0.735	(-0.941, -0.528)	-0.169	(-0.203, -0.135)
Glucose-lowering Med.	1.611	(-0.108, 3.330)	-0.550	(-0.839, -0.261)
Blood pressure-lowering Med.	-3.837	(-5.473, -2.200)	0.207	(-0.082, 0.496)
Lipid-lowering Med.	-1.400	(-2.584, -0.216)	0.349	(0.146, 0.552)

© 2023 Gregorich M et al. *JAMA Network Open*.

eAppendix 7. Partial R^2 Coefficients

The partial relative importance of each predictor was quantified by the drop in the conditional and the marginal R^2 of the full model compared to the reduced model in which the respective predictor was removed from the full model. The partial R^2 (conditional and marginal) is illustrated for each predictor in Figure S4.

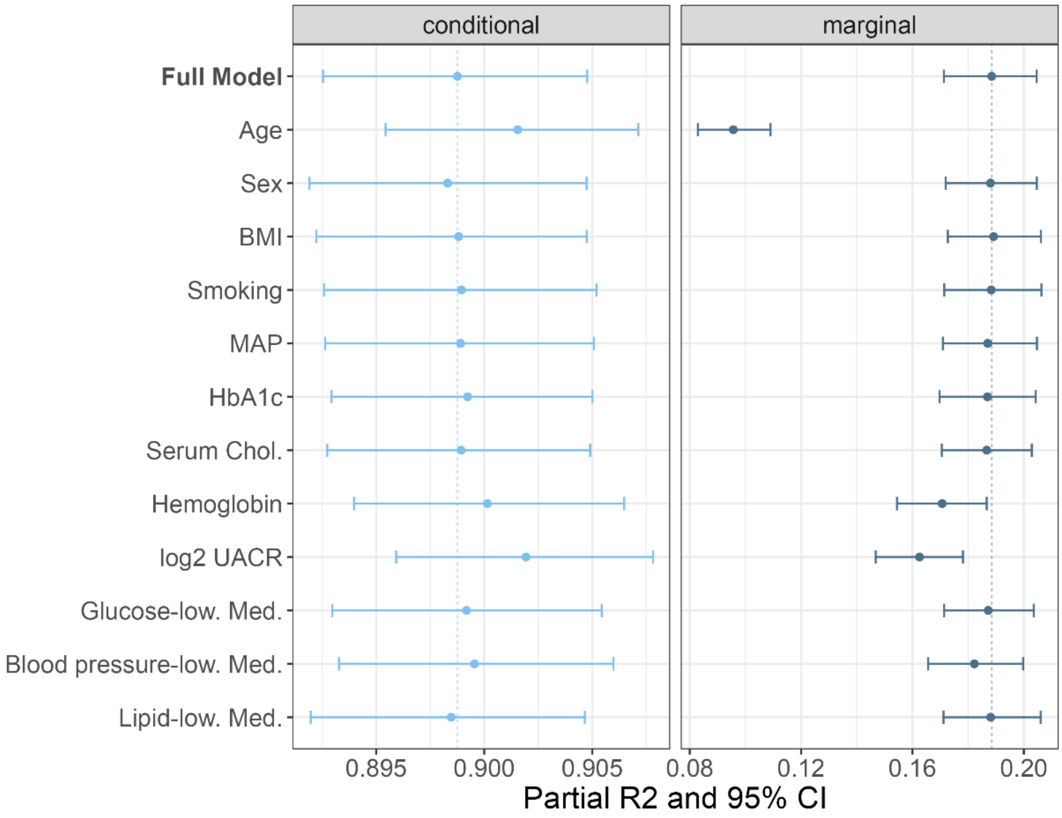

Figure S4. Relative partial importance of each predictor in terms of conditional and marginal R^2 of the reduced model

© 2023 Gregorich M et al. *JAMA Network Open*.

eAppendix 8. Model Comparison

In addition, we specified two additional models to examine whether the number of predictors included into the main model (full model specification) is warranted.

In the first simplified model specification (clinical+eGFR model specification), the following predictors were included into the clinical prediction model: time of follow-up, age, sex (female/male), smoking status (never/ever), BMI (kg/m), mean arterial pressure, blood pressure-lowering medication, glucose- lowering medication and lipid-lowering medication. The outcome remained the longitudinal eGFR measurements and baseline eGFR was again used to estimate more accurate random effect estimates for a new individual.

Laboratory variables such as HbA_{1c}, $\log_2$ UACR, hemoglobin and serum cholesterol were excluded to obtain a simpler model which only relies on clinical variables.

The prior prediction $\hat{Y}_{it} := (\widehat{eGFR})_{it}$ for the new subject i at time t can be obtained by

$$\begin{aligned}\hat{Y}_{it} = {} & 131.337 - 0.882 * X_{time} - 0.821 * X_{age} - 2.932 * [X_{sex=female}] + 0.036 * X_{BMI} + 0.353 * [X_{smoking=ever}] + 0.065 \\ & * X_{MAP} + 0.513 * X_{MedDM} - 4.621 * X_{MedBP} - 1.428 * X_{MedLipid} + X_{time} * (0.003 * X_{age} - 0.298 \\ & * [X_{sex=female}] + 0.003 * X_{BMI} - 0.257 * [X_{smoking=ever}] - 0.022 * X_{MAP} - 0.573 * X_{MedDM} - 0.054 \\ & * X_{MedBP} + 0.184 * X_{MedLipid})\end{aligned}$$

Overall, the exclusion of the simpler model did not significantly impact the performance of the model. However, the exclusion of the laboratory measurements leads to a strong drop in the marginal R^2 of 0.12 and a constant conditional R^2 of 0.9. The performance measures only showed minor changes in the second decimal place.

In the second model specification (model with only UACR), the following predictors were included into the clinical prediction model: time of follow-up and $\log_2$ UACR. All other variables were excluded from the model. The outcome remained the longitudinal eGFR measurements with baseline eGFR being used for updating the random effect estimates for a new observation. The model achieves a marginal R^2 of 0.09 and a conditional R^2 of 0.9. Since, the measures of model performance only slightly decreased compared to Table S4, we will refrain from listing them here.

© 2023 Gregorich M et al. *JAMA Network Open*.

Table S4. Cross-validated and external measures of model performance and validity such as the updated R^2, C statistic and the calibration slope (CS) across follow-up visits for the clinical+eGFR model

	Performance measures					
Follow-up year	*R^2 (95% CI)*		*C statistic (95% CI)*		*CS (95% CI)*	
			Cross-validated			
1	*0.74*	*(0.59, 0.84)*	*0.84*	*(0.78, 0.88)*	*1.06*	*(0.88, 1.16)*
2	*0.62*	*(0.50, 0.76)*	*0.80*	*(0.76, 0.85)*	*1.01*	*(0.85, 1.18)*
3	*0.58*	*(0.42, 0.75)*	*0.80*	*(0.74, 0.85)*	*0.97*	*(0.74, 1.22)*
4	*0.54*	*(0.38, 0.72)*	*0.77*	*(0.71, 0.83)*	*0.98*	*(0.72, 1.26)*
5	*0.47*	*(0.24, 0.67)*	*0.75*	*(0.67, 0.82)*	*0.92*	*(0.60, 1.20)*
			Externally validated			
1[a]	-	-	-	-	-	-
2	*0.69*	*(0.62, 0.76)*	*0.83*	*(0.81, 0.85)*	*1.08*	*(1.02, 1.16)*
3	*0.63*	*(0.57, 0.69)*	*0.82*	*(0.80, 0.83)*	*1.09*	*(1.04, 1.14)*
4	*0.64*	*(0.57, 0.72)*	*0.80*	*(0.78, 0.82)*	*1.13*	*(1.05, 1.20)*
5	*0.57*	*(0.52, 0.61)*	*0.78*	*(0.77, 0.80)*	*1.07*	*(1.01, 1.12)*

However, we compared the models (full model, clinical+eGFR model, UACR only model) using the Akaike Information Criterion (AIC) and the Bayesian Information Criterion (BIC) to determine if the increased complexity of the additional variables in the full model was statistically justified. The results, shown in Table S5, indicate that both the AIC and BIC of the full model are the lowest, indicating the best fit for the data with the full model. In addition, the p-value of the performed log-likelihood ratio test for model comparison is <0.001, indicating that the improvement given by the full model is statistically significant. Thus, the full model should be preferred.

© 2023 Gregorich M et al. *JAMA Network Open*.

Table S5. Model comparison between the full model, the model with the clinical covariates and the model with UACR only as a covariate.

Model	npar	AIC	BIC	Chisq	DF	p
**UACR only**	9	123037	123106			
**Clinical**	23	122295	122471	770.60	14	*<0.001*
**Full**	31	121962	122200	348.41	8	*<0.001*

*npar, number of parameter; AIC, Akaike's information criterion; BIC, Bayesian information criterion; Chisq, Chisquare test statistic; DF, degrees of freedom;

© 2023 Gregorich M et al. *JAMA Network Open*.

eAppendix 9. Composition of Medication Classes

Medication classes were incorporated as binary variables (intake: yes/no) into the model due to the lack of information on individual medications per patient in the GCKD and DIACORE study cohorts. The complete list of drugs included in each medication class can be found in Table S4 for the PROVALID cohort.

Table S6. Composition of the blood-pressure, glucose and lipid lowering medication classes in the PROVALID cohort

Medication class	Drugs
Blood-pressure lowering	ACE inhibitors, Renin inhibitors, Angiotensin-II- receptor blockers, Beta-receptor blockers, Calcium-antagonists, Centrally acting antihypertensives, Alpha-receptor blockers, Direct vasodilators, Loop diuretics, Thiazides, Potassium saving diuretics, Aldosterone antagonists
Glucose lowering	Biguanides (metformin), Insulines, Sulfonylureas, DPPIV inhibitors or GLP1 analogs, Meglitinides (glinides), Thiazolinediones (glitazones), Alpha-Glucosidase-inhibitors, SGLT2 inhibitors (gliflozins)
Lipid lowering	Clofibric acid derivative, Statins, others (ezetimibe, omega 3 acid)

© 2023 Gregorich M et al. *JAMA Network Open*.

eReferences

1. Commenges D, Jacqmin-Gadda H. *Dynamical biostatistical models.* CRC Press; 2015.
2. Verbeke G, Molenberghs G. Linear mixed models for longitudinal data. In: *Linear mixed models in practice.* Springer; 2000.
3. Steyerberg EW, Harrell Jr FE. Prediction models need appropriate internal, internal-external, and external validation. *Journal of Clinical Epidemiology.* 2016;69:245.
4. Steyerberg EW. Validation in prediction research: the waste by data splitting. *Journal of Clinical Epidemiology.* 2018;103:131-133.
5. Steyerberg EW, Uno H, Ioannidis JP, et al. Poor performance of clinical prediction models: the harm of commonly applied methods. *Journal of Clinical Epidemiology.* 2018;98:133-143.
6. Collins GS, Ogundimu EO, Altman DG. Sample size considerations for the external validation of a multivariable prognostic model: a resampling study. *Statistics in Medicine.* 2016;35(2):214-226.

© 2023 Gregorich M et al. *JAMA Network Open*.
