# Supplementary material for: Development and Validation of a Prediction Model for Future Estimated Glomerular Filtration Rate in People With Type 2 Diabetes and Chronic Kidney Disease
Source: JAMA Netw Open. 2023 Apr 5;6(4):e231870. doi: 10.1001/jamanetworkopen.2023.1870 (PMC10077108; doi:10.1001/jamanetworkopen.2023.1870)
Supplement: Supplement 2. — Data Sharing Statement [file jamanetwopen-e231870-s002.pdf]

Data Sharing Statement

Gregorich. Development and Validation of a Prediction Model for Future Estimated Glomerular Filtration Rate in People With Type 2 Diabetes and Chronic Kidney Disease. *JAMA Netw Open*. Published April 05, 2023. doi:10.1001/jamanetworkopen.2023.1870

Data
Data available: No
